# P01-19 Building a cross-sectoral collaboration to support insight, intelligence and innovation for physical activity promotion in Ireland

**DOI:** 10.1093/eurpub/ckac095.019

**Published:** 2022-08-29

**Authors:** Joey Murphy

**Affiliations:** Centre for Exercise, Nutrition and Health Sciences, School for Policy Studies, Bristol

**Keywords:** Collaboration, partnership, engagement

## Abstract

**Issue:**

The national physical activity plan (NPAP) for Ireland originated from an interdepartmental, cross-disciplinary structure that was put in place to promote population physical activity (PA) levels. There is a documented gap between research, policy and practice which hinders this promotion of population levels of PA. The Irish Physical Activity Research Collaboration (I-PARC) was established to play a key role in contributing to the outcomes of the NPAP, including the creation of a platform that enables knowledge translation and the sharing of valuable insight.

**Description of Methods:**

Using elements of participatory action research, the collaboration encompasses 1) cross-sectoral buy in and interaction to reflect upon and understand the current PA landscape in Ireland, 2) a knowledge translation plan that provides strategies for ensuring any collaborative outputs are effectively shared with those relevant, and 3) interaction with PA professionals to generate a common aim and objectives for I-PARC, and identify the added value of sustaining such a collaboration.

**Results:**

To date, I-PARC has gained buy in from key stakeholders (N = 20) involved in PA promotion across government departments (N = 3), government agencies (N = 5) and research institutes (N = 4) in Ireland. Furthermore, the collaboration encompasses a Practitioner Advisory Group (PAG; N = 25), a Research Advisory Panel (RAP; N = 5) and I-PARC members (N = 140). This collaboration has generated a common aim and objectives that are reinforced through a website, social media account and I-PARC led events. Feedback from events and from focus groups with the PAG shows that a cross-sectoral collaboration, such as I-PARC, is needed to support insight, intelligence and innovation to enable more people to be more active in Ireland.

**Lessons:**

Key learnings show that generating buy in from key stakeholders, creating and reinforcing a common aim, use of a knowledge translation plan, and identifying the added value and need for a collaboration have aided with establishing and sustaining I-PARC. Buy in from government departments, agencies and research institutes enables future planning around the sustainability of the collaboration, which will allow for continued collaboration between key stakeholders, narrowing the gap between practice, policy and research, and help work towards the common goal of PA promotion.

